# Anonymous nuclear markers data supporting species tree phylogeny and divergence time estimates in a cactus species complex in South America

**DOI:** 10.1016/j.dib.2015.12.002

**Published:** 2015-12-15

**Authors:** Manolo F. Perez, Bryan C. Carstens, Gustavo L. Rodrigues, Evandro M. Moraes

**Affiliations:** a Departamento de Biologia, Universidade Federal de São Carlos, Rodovia João Leme dos Santos km 110, Sorocaba, São Paulo 18052780, Brazil; b Department of Evolution, Ecology, and Organismal Biology, The Ohio State University, Columbus, OH, USA

**Keywords:** Species tree, Next generation sequencing, Molecular markers, Phylogeography, Non-model species

## Abstract

Supportive data related to the article “Anonymous nuclear markers reveal taxonomic incongruence and long-term disjunction in a cactus species complex with continental-island distribution in South America” (Perez et al., 2016) [1]. Here, we present pyrosequencing results, primer sequences, a cpDNA phylogeny, and a species tree phylogeny.

**Specifications Table**

**Table:** 

Subject area	Biology, Genetics and Genomics
More specific subject area	Phylogenetics and Phylogenomics
Type of data	Pyrosequencing filtering steps, primer sequences and characteristics, species tree analysis input and output, species tree and cpDNA phylogenetic tree
How data was acquired	Pyrosequencing filtering in pyRAD, primer sequences designed with Primer3, primer characteristics gathered with DNAsp, species tree and cpDNA phylogenetic tree generated with BEAST2
Data format	Filtered and analyzed
Experimental factors	n/a
Experimental features	Pyrosequencing of reduced genomic libraries, development of primers and Sanger sequencing for primer validation and missing data reduction
Data source location	n/a
Data accessibility	With this article, GenBank accession numbers GenBank: KU161695–KU162858

## Value of the data

• *Pyrosequencing filtering steps results enable comparisons with other genomic studies in non-model species.*
• Primer sequences allow researchers to test and to use this genomic information in other related taxa.
• Mitochondrial and multilocus phylogenies allow comparing the topologies gathered with the two sets of markers, and also enable comparisons with other codistributed taxa.

## 1 Data

The data shared in this article consist of primer sequences designed after filtering two Pyrosequencing runs, sequencing data from 25 nuclear markers in 40 individuals from 4 species of the *Pilosocereus aurisetus* species complex, and the species tree and chloroplast topologies used in Perez et al. [1].

## 2 Experimental design, materials and methods

### 2.1 Bioinformatic analysis

The Pyrosequencing reads were quality controlled using FASTX-toolkit (http://hannonlab.cshl.edu/fastx_toolkit/) and the pyRAD package [2] to recover variable loci with data available across the species and populations analyzed. The following parameters were applied: (1) ≥5 identical sequences for each allele, to minimize the recovery of sequencing errors and homopolymers; (2) ≤2 different bases for a given nucleotide position, as the organisms are diploid and showed no signal of polyploidy [3]; (3) ≤20 polymorphic sites for each locus, to avoid the inclusion of paralogous loci, that usually show high levels of variation. The remaining dataset after each quality control step is in Table 1. The pyrosequencing data filtering resulted in a total of 223 loci occurring in at least 10 individuals, which were aligned against GenBank with Blastn (Table 2). All loci that matched cytoplasmatic sequences (cp and mtDNA) and retrotransposons were discarded, resulting in 26 loci in all populations sampled. Primers were developed for these loci in the software Primer3 v4.0.0 [4] with the parameters: (1) primer size between 18 and 23 bp; (2) melting (Tm) between 58 and 63 °C; (3) maximum difference of 2 °C for the Tm between forward and reverse primers; GC content of 20–70%. All the developed loci showed specific amplification in at least one sample, but one marker was discarded from further analysis owing to amplification and sequencing problems in the outgroup. Sanger sequencing reactions were obtained for 117 sequences (containing both strands), selected to assure data for at least two individuals for each locus. After combining sequences from both Sanger and pyrosequencing for the 25 loci, a total of 687 sequences over 40 individuals were obtained (Supplementary Table 1), with a total of 367 SNPs. The obtained loci were quality-controlled for recombination using the DSS method [5] as implemented in the software package TOPALi v2 [6], and we also tried to detect loci under selection using Tajima׳s *D*, Fu and Li׳s D* and F* in DNAsp [7]. The results of the quality control for recombination and selection, as well as the main characteristics of each locus are available in Table 3.

### 2.2 Species tree

A species tree was estimated using the STRUCTURE groups (Fig. 1 in Perez et al. [1]) as operational taxonomic units (OTUs) in BEAST 2 [8]. We performed this analysis using a Yule speciation prior, with the most likely model of sequence evolution obtained in jModeltest2 [9]. We used either a strict or a relaxed lognormal clock at each locus, selected after comparing the marginal likelihoods of runs using each model with a Path Sampling analysis with 8 steps and 500,000 generation after a 50% burn-in. The species tree was obtained after two independent runs of 100,000,000 MCMC generations each, with a 10% burn-in, and sampling trees every 5000 steps. The species tree analyzes were performed according to the sequence evolution and clock models recovered for each marker (Table 3). A Maximum Clade Credibility (MCC) tree was generated in TreeAnotator [10], by combining the trees from the two runs. The XML input file, containing all the sequences (also deposited as GenBank accession numbers KU161695–KU162858) used is available in Supplementary data 1. The obtained MCC tree is available in Newick format in the Supplementary data 2.

### 2.3 cpDNA and multiloci data comparison

Comparison of the plastid (partial trnT-trnL and trnS-trnG data from [11]) and the combined multilocus datasets (Fig. 4a in [1]) was performed by contrasting the topology of the species tree analysis with the nuclear data (Supplementary data 2) and the topology of a BEAST phylogenetic analysis with a relaxed lognormal clock in the plastid data. The cpDNA XML file with the sequences is available in Supplementary data 3. The cpDNA tree in Newick format is in Supplementary data 4. The divergence times (Mya) estimate between the two main lineages was also compared (Table 4) by setting them as monophyletic and calculating the time to the Most Recent Common Ancestor (TMRCA) using BEAST for the plastid dataset and the combined multilocus dataset, including the plastid data (Fig. 4b in [1]). Because of the lack of substitution rates for the nuclear markers, relative rates to the plastid marker was used, by using a prior distribution including the minimum and maximum substitution rates observed in the chloroplast sequences of angiosperms [12].

## Figures and Tables

**Table 1:** Results from pyrosequencing runs and filtering steps.

**Filtering step**	**Amount of data**
Total number of reads	2,282,266
Barcoded reads >100 bp and QC	1,511,080
Mean number of aligned loci (95% similarity)	13,218
Mean number of pre-loci (≥ 5 similar sequences in one individual)	892
Paralog filter (≤20 SNPs)	530
Loci in ≥ 10 individuals	223
Loci in all species	167
Loci in all pops	48
Manual paralog inspection	36
Without matches in Genbank	26
Amplified in all individuals tested (including outgroup)	25

**Table 2:** Blastn matches for the pyrosequencing 223 filtered loci occurring in more than 10 individuals.

**Marker type**	**Number of matches**
ANL (*E*-value<10−4)	121
cpDNA	8
mtDNA	27
Retrotransposon	5
RNA	62

**Table 3:** Primers and statistics for each locus.

**Locus**	**Primer Fwd (5′–3′)**	**Primer Rev (5′–3′)**	**Tm**	**N**	**bp**	***S***	***θ***_**w**_	***π***	***D***	***D***⁎	***F***⁎	**Model**	**Strict**	**LogNormal**
PaANL008	TCCTCTCTTTTCTAGGGACGAC	CCCCATTCTTTCTTCATTCTATC	52	58	497	5	0.002	0.001	−0.89	−0.89	−1.04	F81	−776.2717	**−770.3064**
PaANL010	GAGAACGTCAATCCGACAGG	GAACATAGGCTGGCCTCTTC	53	70	473	4	0.002	0.001	−1.03	0.97	0.40	JC	**−738.62**	−743.16
PaANL015	GACCCTAACGAGGGTGAGAC	AAATCATTTCATGAGGCATCG	51	56	461	27	0.020	0.010	−1.57	1.51⁎	0.48	F81+G	−1011.27	**−1002.16**
PaANL017	TGTCCACCCCATAGAAGAGG	TTTAGATGAGTCCCAAAAGATACAC	55	80	309	31	0.020	0.013	−1.11	1.93⁎⁎	0.92	K80	−655.69	**−654.05**
PaANL028	CGTAGCAAACAGACATCCACTT	AAGAAATGCAACAAAAGAGTACCA	54	48	459	13	0.009	0.003	−2.01⁎	0.50	–0.40	F81	−746.59	**−740.17**
PaANL035	TCCTCTTTCCTACCATTCTTTCT	GTTTGAGGAAGGCAGAGGAG	54	44	340	9	0.006	0.002	−1.94⁎	−0.56	−1.18	HKY	−536.95	**−530.30**
PaANL046	ACTTTCCTGTRTCATATGTAA	CGAACTGGCCTCGGATTC	50	48	404	25	0.014	0.006	−1.87⁎	−1.61	−2.02	F81	**−841.24**	−845.07
PaANL050	CGGGTCTAACTTGCCTTCAA	ACCCAACCGGTCAGATTGT	58	52	450	29	0.017	0.016	−0.10	1.27	0.93	HKY+I	−942.70	**−941.18**
PaANL080	AAGAAGAACGGGCGAGTTG	AGGAGGTGGCAATGCAGTAG	58	80	477	25	0.012	0.011	−0.43	1.83⁎⁎	1.18	HKY+G	**−1013.49**	−1013.74
PaANL082	CCAAGCAATATCGCATAAACAA	GGCACTAACTGATTCAATAACTGGT	55	64	383	6	0.003	0.001	−1.72	1.16	0.27	GTR+I	−674.07	**−664.83**
PaANL087	TCTTTATGGCGTTATTCACTCG	CGAAGGCCTAACTTGACAGG	58	46	395	3	0.002	0.001	−1.32	0.90	0.27	K80	−647.56	**−645.56**
PaANL096	AGAAATGTGGGTCAGGAGGA	GAAATGCACATGCCTAGTGA	56	44	436	17	0.011	0.003	−2.18⁎	−2.42	−2.77⁎	F81	−789.03	**−781.24**
PaANL123	TTGCATGTTTATACAATTTTTCTTG	TGATAGATGCCAATCAGTCCAC	55	40	387	18	0.011	0.006	−1.36	1.25	0.45	HKY	−690.90	**−687.04**
PaANL126	TCCTAAACAAGGGCTACGAAG	TGTACCAATGGGCAGCAC	60	52	451	15	0.008	0.005	−1.21	−0.75	−1.07	GTR+I	−901.97	**−893.11**
PaANL134	CGTGGTTTGACAAAACTTACCC	TCAGTGTTTCTAAGATGCTGCAC	58	44	473	17	0.009	0.005	−1.35	−1.21	−1.49	HKY	−837.50	**−830.98**
PaANL140	TAGCCTCCTGAGCCCAAGC	GTTCATCAATGGGGAAGGTG	60	36	478	5	0.003	0.002	−1.45	0.39	−0.20	HKY	−759.99	**−752.42**
PaANL142	CAAGCCTCTCCCTATAAC	TATAGAGTCTAGGCAAGGC	59	36	483	26	0.015	0.013	−0.62	0.41	0.08	K80	−945.42	**−938.42**
PaANL147	CTGTTGGCTCTGCATAGCTG	TGCTACACTGGCTTCATTGC	58	36	440	14	0.010	0.005	−1.60	−0.23	−0.79	F81+G	−940.03	**−922.82**
PaANL155	CTTTTCAGTCCAAAGCAAATTC	AAGGTCAGTAAGTCAAGCTCCTC	56	60	458	5	0.003	0.001	−1.61	1.08	0.27	F81	**−680.40**	−683.15
PaANL160	CGTGCTTTTACCTCCGTAAAG	CTAAGGGCTAATGGTGCTAGG	56	44	489	26	0.014	0.010	−0.93	1.86⁎⁎	0.96	HKY	−839.39	**−838.84**
PaANL165	AGCCCTATATGTGGAAGG	GGAGTGCTTTCAAGCCTTTG	58	38	478	37	0.024	0.013	−1.59	0.62	−0.17	GTR	**−952.36**	−954.68
PaANL182	TTCAGGCTTAGGTTGGTGTTC	AGGGTCGTCACGATCATCC	60	40	476	33	0.019	0.010	−1.68	−2.97⁎	−2.30⁎	HKY	**−945.48**	−945.80
PaANL187	CCGATTGAGGCTAGAAGCTG	TGTCTCTTGGCTTTACTTTAGGG	58	40	485	28	0.015	0.007	−1.92⁎	1.24	0.20	GTR	**−768.93**	−772.03
PaANL196	GCTTGGAGGTTTCCAATGAG	GAATGCTAAGGCCAAAAAGC	56	38	435	43	0.028	0.022	−0.91	1.38	0.70	HKY+I	−818.35	**−816.98**
PaANL205	AAATCGGAGTCACAACAGAGA	TACCGAGATCTTGCGATGC	54	52	382	23	0.013	0.008	−1.46	1.43	0.49	F81	−819.18	**−807.35**

Tm – melting temperature (°C) for each pair of primer, N – number of obtained sequences, bp – length in base pairs, S – number of segregating sites, *θ*w – Waterson׳s theta, π – nucleotide diversity, Tajima׳s *D*, Fu and Li׳s *D*, Fu and Li׳s *F*. Numbers in bold represent the model with higher marginal posterior probabilities after the path sampling test.

⁎ Significance is shown at 0.05.

⁎⁎ Significance is shown at 0.02.

**Table 4:** Comparison of the divergence times (Mya) estimated for the plastid dataset and the combined multilocus dataset.

Parameter	cpDNA	Combined
Mean	1.7027	1.6862
SD	0.5938	0.2515
Variance	0.3526	0.0633
95% HPD	0.6915–2.884	0.9131–1.766

## References

[1] Perez M.F., Carstens B.C., Rodrigues G.L., Moraes E.M. (2016). Anonymous nuclear markers reveal taxonomic incongruence and long-term disjunction in a cactus species complex with continental-island distribution in South America. Mol. Phylogenetics Evol..

[2] Eaton D.A.R. (2014). PyRAD: assembly of de novo RADseq loci for phylogenetic analyses. Bioinformatics.

[3] A.A. Jesus, F.A. Ortolani, E.M. Moraes, Cactaceae, in: K. Marhold (Ed.), IAPT/IOPB Chromosome Data 17, Taxon 63, 2014, 1150–1151 (E12).

[4] Untergrasser A., Cutcutache I., Koressaar T., Ye J., Faircloth B.C., Remm M., Rozen. S.G. (2012). Primer3 – new capabilities and interfaces. Nucleic Acids Res..

[5] McGuire G., Wright F. (2000). TOPALi 2.0: improved detection of mosaic sequences within multiple alignments. Bioinformatics.

[6] Milne I., Lindner D., Bayer M., Husmeier D., McGuire G., Marshall D.F., Wright F. (2009). TOPALi v2: a rich graphical interface for evolutionary analyses of multiple alignments on HPC clusters and multi-core desktops. Bioinformatics.

[7] Librado P., Rozas J. (2009). DnaSP v5: a software for comprehensive analysis of DNA polymorphism data. Bioinformatics.

[8] Bouckaert R., Heled J., Kühnert D., Vaughan T., Wu C.-H., Xie D., Suchard M.A., Rambaut A., Drummond A.J. (2014). BEAST 2: a software platform for bayesian evolutionary analysis. PLoS. Comput. Biol..

[9] Darriba D., Taboada G.L., Doallo R., Posada D. (2012). jModelTest 2: more models, new heuristics and parallel computing. Nat. Met..

[10] Drummond A.J., Rambaut A. (2007). BEAST: Bayesian evolutionary analysis by sampling trees. BMC Evol. Biol..

[11] Bonatelli I.A.S., Perez M.F., Peterson A.T., Taylor N.P., Zappi D.C., Machado M.C., Koch I., Pires A.H.C., Moraes E.M. (2014). Interglacial microrefugia and diversification of a cactus species complex: phylogeography and palaeodistributional reconstructions for *Pilosocereus aurisetus* and allies. Mol. Ecol..

[12] Wolfe K.H., Li W.-H., Harp P.M.S. (1987). Rates of nucleotide substitution vary greatly among plant mitochondrial, chloroplast, and nuclear DNAs. Proc. Natl. Acad. Sci. USA.

