# Supplementary material for: Anonymous nuclear markers data supporting species tree phylogeny and divergence time estimates in a cactus species complex in South America
Source: Data Brief. 2015 Dec 15;6:456–60. doi: 10.1016/j.dib.2015.12.002 (PMC4716445; doi:10.1016/j.dib.2015.12.002)
Supplement: Supplementary file 1 — Supplementary material [file mmc1.docx]

*Data article*

**Title:** Anonymous nuclear markers data supporting species tree phylogeny and divergence time estimates in a cactus species complex in South America.

**Authors:** Perez, MF*; Carstens, BC†; Rodrigues, GL*; Moraes, EM

**Affiliations:** *Departamento de Biologia, Universidade Federal de São Carlos, Rodovia João Leme dos Santos km 110, Sorocaba, São Paulo 18052780, Brazil, †Department of Evolution, Ecology, and Organismal Biology, The Ohio State University, Columbus, OH, USA

**Contact email:** emarsola@ufscar.br

**Abstract**

Supportive data related to the article “Anonymous nuclear markers reveal taxonomic incongruence and long-term disjunction in a cactus species complex with continental-island distribution in South America.” [1]. Here, we present pyrosequencing results, primer sequences, a cpDNA phylogeny, and a species tree phylogeny.

**Specifications Table**

| Subject area | Biology, Genetics and Genomics |
| --- | --- |
| More specific subject area | Phylogenetics and Phylogenomics |
| Type of data | Pyrosequencing filtering steps, primer sequences and characteristics, species tree analysis input and output, species tree and cpDNA phylogenetic tree |
| How data was acquired | Pyrosequencing filtering in pyRAD, primer sequences designed with Primer3, primer characteristics gathered with DNAsp, species tree and cpDNA phylogenetic tree generated with BEAST2 |
| Data format | Filtered and analyzed |
| Experimental factors | n/a |
| Experimental features | Pyrosequencing of reduced genomic libraries, development of primers and Sanger sequencing for primer validation and missing data reduction |
| Data source location | n/a |
| Data accessibility | With this article, GenBank accession numbers KU161695–KU162858 |

**Value of the data**

- *Pyrosequencing filtering steps results enable comparisons with other genomic studies in non-model species.*
- Primer sequences allow researchers to test and to use this genomic information in other related taxa.
- Mitochondrial and multilocus phylogenies allow comparing the topologies gathered with the two sets of markers, and also enable comparisons with other codistributed taxa.

**Data**

The data shared in this article consist of primer sequences designed after filtering two Pyrosequencing runs, sequencing data from 25 nuclear markers in 40 individuals from 4 species of the *Pilosocereus aurisetus* species complex, and the species tree and chloroplast topologies used in Perez et al. [1].

**Experimental Design, Materials and Methods**

*Bioinformatic analysis*

The Pyrosequencing reads were quality controlled using FASTX-toolkit (http://hannonlab.cshl.edu/fastx_toolkit/) and the pyRAD package [2] to recover variable loci with data available across the species and populations analyzed. The following parameters were applied: (1) ≥5 identical sequences for each allele, to minimize the recovery of sequencing errors and homopolymers; (2) ≤2 different bases for a given nucleotide position, as the organisms are diploid and showed no signal of polyploidy [3]; (3) ≤20 polymorphic sites for each locus, to avoid the inclusion of paralogous loci, that usually show high levels of variation. The remaining dataset after each quality control step is in Table 1. The pyrosequencing data filtering resulted in a total of 223 loci occurring in at least 10 individuals, which were aligned against GenBank with Blastn (Table 2). All loci that matched cytoplasmatic sequences (cp and mtDNA) and retrotransposons were discarded, resulting in 26 loci in all populations sampled. Primers were developed for these loci in the software Primer3 v4.0.0 [4] with the parameters: (1) primer size between 18 and 23 bp; (2) melting (Tm) between 58 and 63°C; (3) maximum difference of 2°C for the Tm between forward and reverse primers; GC content of 20-70%. All the developed loci showed specific amplification in at least one sample, but one marker was discarded from further analysis owing to amplification and sequencing problems in the outgroup. Sanger sequencing reactions were obtained for 117 sequences (containing both strands), selected to assure data for at least two individuals for each locus. After combining sequences from both Sanger and pyrosequencing for the 25 loci, a total of 687 sequences over 40 individuals were obtained (Supplementary Table 1), with a total of 367 SNPs. The obtained loci were quality-controlled for recombination using the DSS method [5] as implemented in the software package TOPALi v2 [6], and we also tried to detect loci under selection using Tajima´s D, Fu and Li´s D* and F* in DNAsp [7]. The results of the quality control for recombination and selection, as well as the main characteristics of each locus are available in Table 3.

*Species Tree*

A species tree was estimated using the STRUCTURE groups (Figure 1 in Perez et al. [1]) as operational taxonomic units (OTUs) in BEAST 2 [8]. We performed this analysis using a Yule speciation prior, with the most likely model of sequence evolution obtained in jModeltest2 [9]. We used either a strict or a relaxed lognormal clock at each locus, selected after comparing the marginal likelihoods of runs using each model with a Path Sampling analysis with 8 steps and 500 000 generation after a 50% burn-in. The species tree was obtained after two independent runs of 100 000 000 MCMC generations each, with a 10% burn-in, and sampling trees every 5 000 steps. The species tree analyzes were performed according to the sequence evolution and clock models recovered for each marker (Table 3). A Maximum Clade Credibility (MCC) tree was generated in TreeAnotator [10], by combining the trees from the two runs. The XML input file, containing all the sequences (also deposited as GenBank accession numbers KU161695–KU162858) used is available in Supplementary data 1. The obtained MCC tree is available in Newick format in the Supplementary data 2.

*cpDNA and multiloci data comparison*

Comparison of the plastid (partial trnT-trnL and trnS-trnG data from [11]) and the combined multilocus datasets (Fig 4a in [1]) was performed by contrasting the topology of the species tree analysis with the nuclear data (Supplementary data 2) and the topology of a BEAST phylogenetic analysis with a relaxed lognormal clock in the plastid data. The cpDNA XML file with the sequences is available in Supplementary data 3. The cpDNA tree in Newick format is in Supplementary data 4. The divergence times (Mya) estimate between the two main lineages was also compared (Table 4) by setting them as monophyletic and calculating the time to the Most Recent Common Ancestor (TMRCA) using BEAST for the plastid dataset and the combined multilocus dataset, including the plastid data (Fig4b in [1]). Because of the lack of substitution rates for the nuclear markers, relative rates to the plastid marker was used, by using a prior distribution including the minimum and maximum substitution rates observed in the chloroplast sequences of angiosperms [12].

**References**

[1] M.F. Perez, B.C. Carstens, G.L. Rodrigues, E.M. Moraes, 2016. Anonymous nuclear markers reveal taxonomic incongruence and long-term disjunction in a cactus species complex with continental-island distribution in South America, Mol. Phylogen. Evol. 95, 11-19.

[2] D.A.R. Eaton, 2014. PyRAD: assembly of de novo RADseq loci for phylogenetic analyses, Bioinformatics 30(13), 1844-1849.

[3] A.A. Jesus, F.A. Ortolani, E.M. Moraes, 2014. Cactaceae. In Marhold, K. (ed.), IAPT/IOPB chromosome data 17, Taxon 63, 1150-1151 (E12).

[4] A. Untergrasser, I. Cutcutache, T. Koressaar, J. Ye, B.C. Faircloth, M. Remm, S.G. Rozen., Primer3—new capabilities and interfaces, Nucleic Acids Res. 40 (2012) e115.

[5] G. McGuire, F. Wright, 2000. TOPALi 2.0: Improved Detection of Mosaic Sequences within Multiple Alignments, Bioinformatics 16(2), 130-134.

[6] I. Milne, D. Lindner, M. Bayer, D. Husmeier, G. McGuire, D.F. Marshall, F. Wright, 2009. TOPALi v2: a rich graphical interface for evolutionary analyses of multiple alignments on HPC clusters and multi-core desktops, Bioinformatics 25(1), 126-127.

[7] P. Librado, J. Rozas, 2009. DnaSP v5: A software for comprehensive analysis of DNA polymorphism data, Bioinformatics 25, 1451-1452.

[8] R. Bouckaert, J. Heled, D. Kühnert, T. Vaughan, C-H. Wu, D. Xie, M.A. Suchard, A. Rambaut, A.J. Drummond, 2014. BEAST 2: A Software Platform for Bayesian Evolutionary Analysis, PLoS Comput. Biol. 10(4), e1003537.

[9] D. Darriba, G.L. Taboada, R. Doallo, D. Posada, 2012. jModelTest 2: more models, new heuristics and parallel computing, Nat. Met. 9(8), 772.

[10] A.J. Drummond, A. Rambaut, 2007. BEAST: Bayesian evolutionary analysis by sampling trees, BMC Evol. Biol. 7, 214.

[11] I.A.S. Bonatelli, M.F. Perez, A.T. Peterson, N.P. Taylor, D.C. Zappi, M.C. Machado, I. Koch, A.H.C. Pires, E.M. Moraes, 2014. Interglacial microrefugia and diversification of a cactus species complex: phylogeography and palaeodistributional reconstructions for Pilosocereus aurisetus and allies, Mol. Ecol. 23, 3044–3063.

[12] K.H. Wolfe, W.-H. Li, P.M.S. Harp, 1987. Rates of nucleotide substitution vary greatly among plant mitochondrial, chloroplast, and nuclear DNAs. Proc. Natl. Acad. Sci. USA 84, 9054–9058.
