# Supplementary material for: Anonymous nuclear markers data supporting species tree phylogeny and divergence time estimates in a cactus species complex in South America
Source: Data Brief. 2015 Dec 15;6:456–60. doi: 10.1016/j.dib.2015.12.002 (PMC4716445; doi:10.1016/j.dib.2015.12.002)
Supplement: Supplementary file 2 — Supplementary material [file mmc2.docx]

| Table 1.Results from Pyrosequencing run and filtering steps. | |
| --- | --- |
| **Filtering Step** | **Amount of data** |
| Total number of reads | 2282266 |
| Barcoded reads >100bp and QC | 1511080 |
| Mean number of aligned loci (95% similarity) | 13218 |
| Mean number of pre-loci( ≥ 5 similar sequences in one individual) | 892 |
| Paralog filter (≤20 SNPs) | 530 |
| Loci in ≥ 10 individuals | 223 |
| Loci in all species | 167 |
| Loci in all pops | 48 |
| Manual Paralog inspection | 36 |
| Without matches in Genbank | 26 |
| Amplified in all individuals tested (including outgroup) | 25 |

| Table 2. Blastn matches for the pyrosequencing 223 filtered loci occurring in more than 10 individuals. | |
| --- | --- |
| **Marker type** | **Number of matches** |
| ANL (E-value <10-4) | 121 |
| cpDNA | 8 |
| mtDNA | 27 |
| retrotransposon | 5 |
| RNA | 62 |

| Table 3. Primers and statistics for each locus. Tm - melting temperature (ºC) for each pair of primer, Nsam - number of individuals sequenced times 2, bp - length in base pairs, S - number of segregating sites, θw - Waterson’s theta, π - nucleotide diversity, Tajima’s D, Fu and Li’s D, Fu and Li’s F. Significance is show at 0.05 (*) and 0.02 (**). Numbers in bold represent the model with higher marginal posterior probabilities after the path sampling test. | | | | | | | | | | | | | | |
| --- | --- | --- | --- | --- | --- | --- | --- | --- | --- | --- | --- | --- | --- | --- |
| **Locus** | **Primer Fwd (5´- 3´)** | **Primer Rev (5´- 3´)** | **Tm** | **Nsam** | **bp** | **S** | **θ_w_** | **π** | **D** | **D*** | **F*** | **Model** | **Strict** | **LogNormal** |
| PaANL008 | TCCTCTCTTTTCTAGGGACGAC | CCCCATTCTTTCTTCATTCTATC | 52 | 58 | 497 | 5 | 0.002 | 0.001 | -0.89 | -0.89 | -1.04 | F81 | -776.2717 | **-770.3064** |
| PaANL010 | GAGAACGTCAATCCGACAGG | GAACATAGGCTGGCCTCTTC | 53 | 70 | 473 | 4 | 0.002 | 0.001 | -1.03 | 0.97 | 0.40 | JC | **-738.62** | -743.16 |
| PaANL015 | GACCCTAACGAGGGTGAGAC | AAATCATTTCATGAGGCATCG | 51 | 56 | 461 | 27 | 0.020 | 0.010 | -1.57 | 1.51* | 0.48 | F81+G | -1011.27 | **-1002.16** |
| PaANL017 | TGTCCACCCCATAGAAGAGG | TTTAGATGAGTCCCAAAAGATACAC | 55 | 80 | 309 | 31 | 0.020 | 0.013 | -1.11 | 1.93** | 0.92 | K80 | -655.69 | **-654.05** |
| PaANL028 | CGTAGCAAACAGACATCCACTT | AAGAAATGCAACAAAAGAGTACCA | 54 | 48 | 459 | 13 | 0.009 | 0.003 | -2.01* | 0.50 | -0.40 | F81 | -746.59 | **-740.17** |
| PaANL035 | TCCTCTTTCCTACCATTCTTTCT | GTTTGAGGAAGGCAGAGGAG | 54 | 44 | 340 | 9 | 0.006 | 0.002 | -1.94* | -0.56 | -1.18 | HKY | -536.95 | **-530.30** |
| PaANL046 | ACTTTCCTGTRTCATATGTAA | CGAACTGGCCTCGGATTC | 50 | 48 | 404 | 25 | 0.014 | 0.006 | -1.87* | -1.61 | -2.02 | F81 | **-841.24** | -845.07 |
| PaANL050 | CGGGTCTAACTTGCCTTCAA | ACCCAACCGGTCAGATTGT | 58 | 52 | 450 | 29 | 0.017 | 0.016 | -0.10 | 1.27 | 0.93 | HKY+I | -942.70 | **-941.18** |
| PaANL080 | AAGAAGAACGGGCGAGTTG | AGGAGGTGGCAATGCAGTAG | 58 | 80 | 477 | 25 | 0.012 | 0.011 | -0.43 | 1.83** | 1.18 | HKY+G | **-1013.49** | -1013.74 |
| PaANL082 | CCAAGCAATATCGCATAAACAA | GGCACTAACTGATTCAATAACTGGT | 55 | 64 | 383 | 6 | 0.003 | 0.001 | -1.72 | 1.16 | 0.27 | GTR+I | -674.07 | **-664.83** |
| PaANL087 | TCTTTATGGCGTTATTCACTCG | CGAAGGCCTAACTTGACAGG | 58 | 46 | 395 | 3 | 0.002 | 0.001 | -1.32 | 0.90 | 0.27 | K80 | -647.56 | **-645.56** |
| PaANL096 | AGAAATGTGGGTCAGGAGGA | GAAATGCACATGCCTAGTGA | 56 | 44 | 436 | 17 | 0.011 | 0.003 | -2.18* | -2.42 | -2.77* | F81 | -789.03 | **-781.24** |
| PaANL123 | TTGCATGTTTATACAATTTTTCTTG | TGATAGATGCCAATCAGTCCAC | 55 | 40 | 387 | 18 | 0.011 | 0.006 | -1.36 | 1.25 | 0.45 | HKY | -690.90 | **-687.04** |
| PaANL126 | TCCTAAACAAGGGCTACGAAG | TGTACCAATGGGCAGCAC | 60 | 52 | 451 | 15 | 0.008 | 0.005 | -1.21 | -0.75 | -1.07 | GTR+I | -901.97 | **-893.11** |
| PaANL134 | CGTGGTTTGACAAAACTTACCC | TCAGTGTTTCTAAGATGCTGCAC | 58 | 44 | 473 | 17 | 0.009 | 0.005 | -1.35 | -1.21 | -1.49 | HKY | -837.50 | **-830.98** |
| PaANL140 | TAGCCTCCTGAGCCCAAGC | GTTCATCAATGGGGAAGGTG | 60 | 36 | 478 | 5 | 0.003 | 0.002 | -1.45 | 0.39 | -0.20 | HKY | -759.99 | **-752.42** |
| PaANL142 | CAAGCCTCTCCCTATAAC | TATAGAGTCTAGGCAAGGC | 59 | 36 | 483 | 26 | 0.015 | 0.013 | -0.62 | 0.41 | 0.08 | K80 | -945.42 | **-938.42** |
| PaANL147 | CTGTTGGCTCTGCATAGCTG | TGCTACACTGGCTTCATTGC | 58 | 36 | 440 | 14 | 0.010 | 0.005 | -1.60 | -0.23 | -0.79 | F81+G | -940.03 | **-922.82** |
| PaANL155 | CTTTTCAGTCCAAAGCAAATTC | AAGGTCAGTAAGTCAAGCTCCTC | 56 | 60 | 458 | 5 | 0.003 | 0.001 | -1.61 | 1.08 | 0.27 | F81 | **-680.40** | -683.15 |
| PaANL160 | CGTGCTTTTACCTCCGTAAAG | CTAAGGGCTAATGGTGCTAGG | 56 | 44 | 489 | 26 | 0.014 | 0.010 | -0.93 | 1.86** | 0.96 | HKY | -839.39 | **-838.84** |
| PaANL165 | AGCCCTATATGTGGAAGG | GGAGTGCTTTCAAGCCTTTG | 58 | 38 | 478 | 37 | 0.024 | 0.013 | -1.59 | 0.62 | -0.17 | GTR | **-952.36** | -954.68 |
| PaANL182 | TTCAGGCTTAGGTTGGTGTTC | AGGGTCGTCACGATCATCC | 60 | 40 | 476 | 33 | 0.019 | 0.010 | -1.68 | -2.97* | -2.30* | HKY | **-945.48** | -945.80 |
| PaANL187 | CCGATTGAGGCTAGAAGCTG | TGTCTCTTGGCTTTACTTTAGGG | 58 | 40 | 485 | 28 | 0.015 | 0.007 | -1.92* | 1.24 | 0.20 | GTR | **-768.93** | -772.03 |
| PaANL196 | GCTTGGAGGTTTCCAATGAG | GAATGCTAAGGCCAAAAAGC | 56 | 38 | 435 | 43 | 0.028 | 0.022 | -0.91 | 1.38 | 0.70 | HKY+I | -818.35 | **-816.98** |
| PaANL205 | AAATCGGAGTCACAACAGAGA | TACCGAGATCTTGCGATGC | 54 | 52 | 382 | 23 | 0.013 | 0.008 | -1.46 | 1.43 | 0.49 | F81 | -819.18 | **-807.35** |

| Table 4. Comparison of the divergence times (Mya) estimated for the plastid dataset and the combined multilocus dataset. | | |
| --- | --- | --- |
| Parameter | cpDNA | Combined |
| Mean | 1,7027 | 1,6862 |
| SD | 0,5938 | 0,2515 |
| Variance | 0,3526 | 0,0633 |
| 95% HPD | 0.6915-2.884 | 0.9131-1.766 |

| Supplementary Table 1. Schematic representation of the available sequences on the utilized dataset. Sequences obtained from the raw pyrosequencing data are represented by blue cells and data obtained by Sanger sequencing is represented by green cells. Missing data is represented by white cells. Population codes follow Table 1 and Figure 1 in Perez et al. [1]. | | | | | | | | | | | | | | | | | | | | | | | | | | |
| --- | --- | --- | --- | --- | --- | --- | --- | --- | --- | --- | --- | --- | --- | --- | --- | --- | --- | --- | --- | --- | --- | --- | --- | --- | --- | --- |
|  |  | **Locus (PaANL)** | | | | | | | | | | | | | | | | | | | | | | | | |
| Population | Individual | 8 | 10 | 15 | 17 | 28 | 35 | 46 | 50 | 80 | 82 | 87 | 96 | 123 | 126 | 134 | 140 | 142 | 147 | 155 | 160 | 165 | 182 | 187 | 196 | 205 |
| GMO | GMO-1 |  |  |  |  |  |  |  |  |  |  |  |  |  |  |  |  |  |  |  |  |  |  |  |  |  |
|  | GMO-2 |  |  |  |  |  |  |  |  |  |  |  |  |  |  |  |  |  |  |  |  |  |  |  |  |  |
|  | GMO-3 |  |  |  |  |  |  |  |  |  |  |  |  |  |  |  |  |  |  |  |  |  |  |  |  |  |
|  | GMO-4 |  |  |  |  |  |  |  |  |  |  |  |  |  |  |  |  |  |  |  |  |  |  |  |  |  |
|  | GMO-5 |  |  |  |  |  |  |  |  |  |  |  |  |  |  |  |  |  |  |  |  |  |  |  |  |  |
| MEN | MEN-1 |  |  |  |  |  |  |  |  |  |  |  |  |  |  |  |  |  |  |  |  |  |  |  |  |  |
|  | MEN-2 |  |  |  |  |  |  |  |  |  |  |  |  |  |  |  |  |  |  |  |  |  |  |  |  |  |
|  | MEN-3 |  |  |  |  |  |  |  |  |  |  |  |  |  |  |  |  |  |  |  |  |  |  |  |  |  |
|  | MEN-4 |  |  |  |  |  |  |  |  |  |  |  |  |  |  |  |  |  |  |  |  |  |  |  |  |  |
|  | MEN-5 |  |  |  |  |  |  |  |  |  |  |  |  |  |  |  |  |  |  |  |  |  |  |  |  |  |
| RVE | RVE-1 |  |  |  |  |  |  |  |  |  |  |  |  |  |  |  |  |  |  |  |  |  |  |  |  |  |
|  | RVE-2 |  |  |  |  |  |  |  |  |  |  |  |  |  |  |  |  |  |  |  |  |  |  |  |  |  |
|  | RVE-3 |  |  |  |  |  |  |  |  |  |  |  |  |  |  |  |  |  |  |  |  |  |  |  |  |  |
|  | RVE-4 |  |  |  |  |  |  |  |  |  |  |  |  |  |  |  |  |  |  |  |  |  |  |  |  |  |
| ART | ART-1 |  |  |  |  |  |  |  |  |  |  |  |  |  |  |  |  |  |  |  |  |  |  |  |  |  |
|  | ART-2 |  |  |  |  |  |  |  |  |  |  |  |  |  |  |  |  |  |  |  |  |  |  |  |  |  |
|  | ART-3 |  |  |  |  |  |  |  |  |  |  |  |  |  |  |  |  |  |  |  |  |  |  |  |  |  |
|  | ART-4 |  |  |  |  |  |  |  |  |  |  |  |  |  |  |  |  |  |  |  |  |  |  |  |  |  |
|  | ART-5 |  |  |  |  |  |  |  |  |  |  |  |  |  |  |  |  |  |  |  |  |  |  |  |  |  |
| APA | APA-1 |  |  |  |  |  |  |  |  |  |  |  |  |  |  |  |  |  |  |  |  |  |  |  |  |  |
|  | APA-2 |  |  |  |  |  |  |  |  |  |  |  |  |  |  |  |  |  |  |  |  |  |  |  |  |  |
|  | APA-3 |  |  |  |  |  |  |  |  |  |  |  |  |  |  |  |  |  |  |  |  |  |  |  |  |  |
|  | APA-4 |  |  |  |  |  |  |  |  |  |  |  |  |  |  |  |  |  |  |  |  |  |  |  |  |  |
|  | APA-5 |  |  |  |  |  |  |  |  |  |  |  |  |  |  |  |  |  |  |  |  |  |  |  |  |  |
| DEL | DEL-1 |  |  |  |  |  |  |  |  |  |  |  |  |  |  |  |  |  |  |  |  |  |  |  |  |  |
|  | DEL-2 |  |  |  |  |  |  |  |  |  |  |  |  |  |  |  |  |  |  |  |  |  |  |  |  |  |
|  | DEL-3 |  |  |  |  |  |  |  |  |  |  |  |  |  |  |  |  |  |  |  |  |  |  |  |  |  |
|  | DEL-4 |  |  |  |  |  |  |  |  |  |  |  |  |  |  |  |  |  |  |  |  |  |  |  |  |  |
|  | DEL-5 |  |  |  |  |  |  |  |  |  |  |  |  |  |  |  |  |  |  |  |  |  |  |  |  |  |
| CRI | CRI-1 |  |  |  |  |  |  |  |  |  |  |  |  |  |  |  |  |  |  |  |  |  |  |  |  |  |
|  | CRI-2 |  |  |  |  |  |  |  |  |  |  |  |  |  |  |  |  |  |  |  |  |  |  |  |  |  |
|  | CRI-3 |  |  |  |  |  |  |  |  |  |  |  |  |  |  |  |  |  |  |  |  |  |  |  |  |  |
|  | CRI-4 |  |  |  |  |  |  |  |  |  |  |  |  |  |  |  |  |  |  |  |  |  |  |  |  |  |
|  | CRI-5 |  |  |  |  |  |  |  |  |  |  |  |  |  |  |  |  |  |  |  |  |  |  |  |  |  |
| PIR | PIR-1 |  |  |  |  |  |  |  |  |  |  |  |  |  |  |  |  |  |  |  |  |  |  |  |  |  |
|  | PIR-2 |  |  |  |  |  |  |  |  |  |  |  |  |  |  |  |  |  |  |  |  |  |  |  |  |  |
|  | PIR-3 |  |  |  |  |  |  |  |  |  |  |  |  |  |  |  |  |  |  |  |  |  |  |  |  |  |
|  | PIR-4 |  |  |  |  |  |  |  |  |  |  |  |  |  |  |  |  |  |  |  |  |  |  |  |  |  |
|  | PIR-5 |  |  |  |  |  |  |  |  |  |  |  |  |  |  |  |  |  |  |  |  |  |  |  |  |  |
| Outgroup | *P.Gou*-1 |  |  |  |  |  |  |  |  |  |  |  |  |  |  |  |  |  |  |  |  |  |  |  |  |  |
